# Metabolic and physiological changes induced by plant growth regulators and plant growth promoting rhizobacteria and their impact on drought tolerance in *Cicer arietinum L*.

**DOI:** 10.1371/journal.pone.0213040

**Published:** 2019-03-04

**Authors:** Naeem Khan, Asghari Bano, MD Ali Babar

**Affiliations:** 1 Department of Plant Sciences, Quaid-i-Azam University, Islamabad, Pakistan; 2 Department of Biosciences, University of Wah, Wah Cantt., Pakistan; 3 Department of Agronomy, University of Florida, Gainesville, Florida, United States of America; Louisiana State University College of Agriculture, UNITED STATES

## Abstract

Plant growth regulators (PGRs) and plant growth promoting rhizobacteria (PGPRs) play an important role in mitigating abiotic stresses. However, little is known about the parallel changes in physiological processes coupled with metabolic changes induced by PGRs and PGPRs that help to cope with drought stress in chickpeas. The present investigation was carried out to study the integrative effects of PGRs and PGPRs on the physiological and metabolic changes, and their association with drought tolerance in two chickpea genotypes. Inoculated seeds of two chickpea genotypes, Punjab Noor-2009 (drought sensitive) and 93127 (drought tolerance), were planted in greenhouse condition at the University of Florida. Prior to sowing, seeds of two chickpea varieties were soaked for 3 h in 24 h old cultures of PGPRs (*Bacillus subtilis*, *Bacillus thuringiensis*, and *Bacillus megaterium*), whereas, some of the seeds were soaked in distilled water for the same period of time and were treated as control. Plant growth regulators, salicylic acid (SA) and putrescine (Put), were applied on 25 days old seedlings just prior to the induction of drought stress. Drought stress was imposed by withholding the supply of water on 25-day-old seedlings (at the three-leaf stage) and continued for the next 25 days until the soil water content reached 14%. Ultrahigh-performance liquid chromatography-high resolution mass spectrometry (UPLC-HRMS) analysis concomitant with physiological parameters were carried out in chickpea leaves at two-time points i.e. 14 and 25 d after imposition of drought stress. The results showed that both genotypes, treated with PGRs and PGPRs (consortium), performed significantly better under drought condition through enhanced leaf relative water content (RWC), greater biomass of shoot and root, higher Fv/FM ratio and higher accumulation of protein, sugar and phenolic compounds. The sensitive genotype was more responsive than tolerant one. The results revealed that the accumulation of succinate, leucine, disaccharide, saccharic acid and glyceric acid was consistently higher in both genotypes at both time points due to PGRs and PGPRs treatment. Significant accumulation of malonate, 5-oxo-L-proline, and trans-cinnamate occurred at both time points only in the tolerant genotype following the consortium treatment. Aminoacyl-tRNA, primary and secondary metabolite biosynthesis, amino acid metabolism or synthesis pathways, and energy cycle were significantly altered due to PGRs and PGPRs treatment. It is inferred that changes in different physiological and metabolic parameters induced by PGRs and PGPRs treatment could confer drought tolerance in chickpeas.

## Introduction

Chickpea (*Cicer arietinum* L.) is a legume belongs in the family) Fabaceae, subfamily Faboideae. It is one of the most widely consumed pulse legumes and ranking third after peas and soybean, and also covers a total of 15% of the world’s pulse productions [1]. It is an important source of protein, carbohydrate, B-group vitamins, and different minerals [2]. It is considered an important source of cheap protein with high energy and nutritional values [3, 4]. Drought stress is the most prevalent environmental factor that limits growth, survival, and productivity of chickpeas [5]. The yield of chickpeas can be reduced from 15 to 60% due to the drought stress. Moreover, the global climate change including high temperature stress and unpredictable rainfall pattern coupled with the increasing world population is creating immense pressure on our capacity for sustainable food production including chickpea production. Drought affects seed germination and seedling establishment in the field, however, genotypes vary in their capacity to tolerate drought stress. Drought also causes a substantial reduction in crop productivity through negatively impacting plant growth, physiology, nutrient and water relations, photosynthesis, and assimilate partitioning [6–8]. To cope with such challenges and develop stress resilient chickpea varieties for future climate change condition, understanding the effects of drought on physiological, morphological and biochemical processes, and their relationship to the adaptation mechanisms is crucial [9].

Plant growth regulators (PGRs) or hormones are chemical substances that profoundly influence the growth and differentiation of plant cells, tissues, and organs. They function as chemical messengers for intercellular communication [10]. They have been found to improve tolerance of plants against the damages caused by abiotic stresses. However, limited researches have been led to examine the possible benefits of exogenous application of PGRs under water stress conditions [11], particularly in chickpeas. Plant hormones interact with complex signalling networks to balance the responses to developmental and environmental signals, and thus limit defense-associated fitness costs [12]. Empirical evidence suggests that PGRs, such as, salicylic acid (SA) signalling mechanism positively regulates plant defense against biotrophic pathogens, which requires living tissue to complete their life cycle [13, 14]. SA evidenced to provide tolerance in plants against different abiotic stresses, such as heat, salinity, heavy metal toxicity, and drought [15]. Results have also demonstrated that SA improved tolerance of chickpea seedlings to drought stress [16, 17] and also mitigated the adverse effects of Lead and Mercury on membrane damage [18]. Putrescine (Put) also plays a positive role in reducing the adverse effects of abiotic stresses on plants through its acid neutralizing and cell wall stabilizing capabilities [19]. Put has demonstrated the capability to improve tolerance against drought, oxidative, salinity and chilling stresses in different plant species [20–22]. Beside its role in tolerance, PGRs also influence different developmental processes in plants [23].

Plant growth promoting rhizobacteria (PGPRs) playan important role in increasing crop yields by facilitating plant growth through different mechanisms [24]. PGPRs affect plant growth positively through the production of phytohormones, increased phosphorus availability, and expansion of plant root systems to uptake more water and nutrients. In addition, PGPRs also affect enzymatic activities such as ACC-deaminase, production of rhizobiotoxine to reduce the adverse effects of ethylene and enhance nodulation and fixation of atmospheric nitrogen [25]. PGPRs greatly affect soil characteristics as well and play a vital role in transforming the poor quality land into the cultivable land [26]. However, the successful utilization of PGPRs is dependent on its survival in soil, the compatibility with the crop on which it is inoculated, the interaction ability with indigenous microflora in the soil, and with the environmental factors [24]. In addition, some PGPRs may possess some more specific plant growth-promoting traits, such as heavy metal detoxifying activities, salinity tolerance, and biological control of phytopathogens and insects [27].

Different species of PGPRs, such as *Bacillus*, can be found in the agricultural fields that can promote the crop health in different ways. Some of these species directly stimulate plant growth either through enhancing acquisition of nutrients or through stimulating host plant’s defense against insect and pathogen infection [28]. The genetics, biochemistry, and ecology of *Bacillus subtilis*, a species of *Bacillus*, has been described by different authors [29]. This strain produces indole accetic acid (IAA), siderophore, phytase, organic acid, 1-aminocyclopropane-1-carboxylate (ACC) deaminase, cyanogens, lytic enzymes, oxalate oxidase, and solubilized various sources of organic and inorganic phosphates as well as potassium and zinc. *Bacillus subtilis* stimulates production of phytohormones involved in metabolism and growth development mechanisms [30]. *Bacillus thuringiensis* has been used as an effective bioinsecticide because it produces the proteins Cry and Cyt, which are highly toxic to insects [31]. More recent studies suggest that *B*. *thuringiensis* can be used as a biological control agent to suppress plant disease, and to promote plant growth, seed germination and shoot elongation [32]. *Bacillus megaterium* influences plant growth and development by producing phytohormones such as auxins, gibberellins, and cytokinins [33].

Both PGPRs and PGRs exert beneficial effects on plant growth when applied alone, however, their combined applications were much more effective than PGPRs and/or PGRs used alone to mitigate drought stress in chickpea and wheat. Addition of PGRs to PGPRs inoculated plants assisted in osmoregulation and ameliorated oxidative stresses and induced new proteins, and significantly enhanced the leaf chlorophyll and sugar content. Combined application of PGRs and PGPRs decreased lipid peroxidation more effectively and increased the leaf area. The relative water content in leaves, and root fresh and dry weight were also higher in combined treatment of PGPRs and PGRs. The nutrient content of rhizosphere soil of PGPRs and PGRs treated plants was also enhanced significantly as compared to single application of PGPRs and PGRs. It is inferred from our previous studies that PGPRs and PGRs work synergistically to promote growth of plants under moisture and nutrient deficit condition [17, 34].

A metabolome is a complete set of metabolites produced by an organism in its lifetime. The metabolites play key roles in the biochemical processes of organisms [35]. Metabolomics is one of the fastest growing technologies to understand biochemical changes associated with stress tolerance in plants. This technology is utilized in plant research and includes metabolic fingerprinting, profiling and targeted analysis. Metabolomics is used as an important tool to understand the environmental responses in plants [36]. Empirical evidence suggests that metabolic components are linked to high-temperature stress tolerance in corn [37], in cool season grass [38], and in wheat [39]; drought tolerance in wheat [40, 41], in chickpeas [42], and in rice [43]. Plants can modify their physiology to adapt to different environmental conditions through metabolic changes [44, 45]. Our previous study clearly demonstrated that the combination of PGRs and PGPRs was more effective in ameliorating drought stress in chickpea compared to PGPRs or PGRs alone [17, 42]. In our previous study, we also presented altered metabolic states in two chickpea genotypes, Punjab Noor-2009 (drought sensitive) and 93127 (drought tolerance), under drought stress conditions [34]. Though the combination of PGRs and PGPRs can effectively contribute to the drought tolerance, no known information is available on the complex metabolic regulation associated with PGRs and PGPRs, and their effect on drought stress tolerance in chickpeas. Metabolic profiling can help us to understand the biochemical mechanism involved with PGRs and PGPRs induced drought tolerance in chickpeas and can contribute in developing stress resilient variety development for future food security. Therefore, the present study was carried out to investigate the combined effects of PGRs/PGPRs on the metabolic profiling of two different chickpea genotypes, contrasting for drought tolerance, grown under drought condition. This study also investigated the relationship between altered metabolic levels with different physiological traits under drought stress condition.

## Materials and methods

The experiment was conducted under greenhouse condition at the Department of Agronomy, University of Florida, Gainesville, Florida in May, 2016. Seeds of two chickpea genotypes, Punjab Noor-2009 (drought sensitive) and 93127 (drought tolerance), were obtained from Ayub Agriculture Research Institute, Faisalabad, Pakistan. Initially, chickpea seeds were washed in distilled water followed by surface sterilization with 95% ethanol for 2–3 min and then soaked in 10% Clorox with concomitant shaking. The seeds were subsequently washed in autoclaved distilled water. Some of the washed seeds were soaked for 3 h, prior to sowing, in 24 h old cultures of *Bacillus subtilis*, *Bacillus thuringiensis*, and *Bacillus megaterium*, whereas, some of the washed seeds were sown without any treatment (drought control). The PGRs, salicylic acid (SA) and putrescine (Put) were sprayed (150 mg/L) on 25 days old seedlings of chickpea inoculated with PGPRs. Seeds were grown in pots (5 seeds/pot) measuring 30 × 40 cm^2^ and filled with 2,000 g of Metro-Mix 360 soil mixture. The pots were well watered (twice per week) throughout until drought stress was applied, and each pot contained 5 plants. Water was applied until the soil mix was completely wet and the water started to seep out through the holes at the bottom of the pot. A teaspoon of Osmocote (15N–9P–12K) was applied one time after germination. The green house condition was maintained at 26 and 19 ± 1 °C (day and night temperatures) with 70 ± 2% relative humidity and 11 and 13 hours day and night lengths, respectively. Drought stress was imposed on 25-day-old plants (at the 3-leaf stage) by withholding water supply for the next 25 days until the soil water content reached 14%. Leaf tissue samples were collected twice; at 14 days (first-time point) and 25 days (second-time point) after drought stress initiated for metabolomics analysis and different physiological trait estimation.

The present experiment comprised 2 different treatments; plants under drought stress treated with 3 PGPRs (*Bacillus subtilis*, *Bacillus thuringiensis*, and *Bacillus megaterium*) and 2 PGRs (salicylic acid and putrescine) designated as “consortium”, and plants under drought stress without PGPRs and PGRs treatment and designated as “drought”. The experiment was laid out in a completely randomized block design with 6 replications.

### Method of inoculation

The Luria Bertani (LB) broth was inoculated with fresh (24 h old) bacterial culture. The inoculated LB broth incubated in a shaker for 24 h at 27 °C followed by centrifugation at 10000 rpm (10 min). The pellet was mixed with distilled water and the optical density (OD) (at 660 nm) was adjusted to 1. The colony was then soaked in broth with isolates. The seeds were soaked in broth for 3 h prior to sowing.

### Leaf chlorophyll content and chlorophyll fluorescence

Leaf chlorophyll content was determine by using SPAD chlorophyll meter (SPAD-502 plus. serial No. 20001472 made by Konica Minolta, Japan). Chlorophyll fluorescence was measured on intact leaves of the abaxial surface (third leaf) after 30 min of dark adaptation with a pulse modular fluorometer (Model OS5-FL, Opti- Sciences, Hudson, NH). Chlorophyll fluorescence and chlorophyll content were measured on 3 leaflets in each plant and 5 plants per pot (a total of 15 readings) and averaged. The average value of 15 readings was considered as a single replication, and 6 replicated values/variety were used for statistical analysis and comparison of treatment means, and significant testing at *P* < .05 level.

### Relative water content (RWC)

The relative water content (RWC) of leaves for each treatment was calculated according to the formula of Weatherly [46].

RWC=[(freshweightofleaves−dryweightofleaves)/(turgidweightofleaves−dryweightofleaves)]×100.

### Leaf protein content

The Lowery et al. [47] method was used for the estimation of protein content in leaves of chickpea. In detail, leaf tissue was ground in a phosphate buffer (1 mL) and centrifuged for 10 min. The supernatant was transferred to a tube and distilled water was added for a final volume of 1 mL. Reagents C and D were added to the supernatant and were mixed by shaking. The sample was then incubated at room temperature for 30 min and the absorbance at 650 nm of each sample was determined along with the absorbance of different concentrations of bovine serum albumin (BSA). Protein concentration was calculated as:

Proteinconcentrationmg/g=Kvalue×Dilutionfactor×Absorbance/samplewt.

K value = 19.6, Dilution factor = 2, Wt. of sample = 0.1 g

### Sugar estimation

Sugar content was estimated as outlined by Dube et al. [48]. Fresh leaf tissue was ground in 10 mL of distilled water and centrifuged at 3000 rpm for 5 min. The supernatant (0.1 mL) was mixed with phenol (1 mL; 80%) and concentrated H_2_SO_4_ (5 mL). Absorbance was recorded at 420 nm of wavelength. The concentration of the unidentified sample was considered with reference to the standard curve made by using glucose:

Sugarconcentrationmg/g=Kvalue×Dilutionfactor×Absorbance/SampleWt.

K value = 20, Dilution factor = 10, Wt. of sample = 0.5 g

### Total phenolic content

The total phenolic content of the extract was determined by the Folin—Ciocalteu method [49]. Briefly, crude extract (200 μL) of 3 mL was made with distilled water, mixed thoroughly with Folin—Ciocalteu reagent (0.5 mL) for 3 min, followed by the addition of 2 mL of 20% (w/v) sodium carbonate. The mixture was kept in dark for one hour, and absorbance was measured at 650 nm. The total phenolic content was calculated from the calibration curve, and the results were expressed as mg of gallic acid equivalent per g dry weight (GAE/g).

### Shoot and root dry weights estimation

Shoots and roots of the same 5 plants per replication were cut at the base and dried at 60 °C for 72 hr, and dry weight was taken by using an electronic scale. The root and shoot dry weights were measured after 25 days of drought stress imposition [42].

### Leaf tissue collection and sample preparation for metabolites

Leaf samples from the consortium and drought-stressed plants were collected at midday for metabolic profiling. Leaves were harvested at 14 days (time point 1) and at 25 days (time point 2) after the imposition of drought stress. The sampled leaf tissues were frozen in liquid nitrogen immediately after collection and stored at -80 °C. Tissue samples were lyophilized for 72 hours and ground using a TissueLyser. Lyophilized powder (30 mg) was used for ultrahigh performance liquid chromatography-high resolution mass spectrometry (UPLC-HRMS) based metabolite profiling following the protocol of Lisec et al. [50].

In brief, freeze-dried leaf tissues were weighed (30 mg) into a clean Eppendorf tube, followed by the addition of internal standards (20 μL) to each sample. Methanol (750 μL) and ammonium acetate (10 mM, 750 μL) was added to each sample and vortexed for 1 min at room temperature. Centrifugation (17000 G, 10 m) was done after all the samples were ultra-sonicated for 20 min at room temperature. The supernatant (> 1 mL) was transferred to a 1.5 mL Eppendorf tube, followed by a 50 μL transfer of supernatant to an Eppendorf tube. The supernatant was dried down after adding 50 μL of injection of standard solution. Samples were then vortexed for 30 sec and put at 4 °C for 10 min, centrifuged at 20,000 rpm for 10 min, and the supernatant was transferred into an LC-vial.

### UPLC—HRMS analysis

Untargeted metabolic profiling was performed on an ultrahigh performance liquid chromatography-high resolution mass spectrometry (Model: Thermo Ultimate 3000 UPLC and Thermo QExactive mass spectrometer) platform at the University of Florida Southeast Center for Integrated Metabolomics (SECIM). All samples were analyzed in positive and negative heated electrospray ionization with a mass resolution of 70,000 at *m/z* 200 as separate injections. Chromatographic separation was attained on an ACE Excel 2 C18 PFP100 × 2.1 mm, a 2 μm particle size column with mobile phase A as 0.1% formic acid in water, and mobile phase B as acetonitrile, at a flow rate of 350 μL/min with a run time of 16.8 min, mass resolution of 35,000 at *m/z* 200, and mass range of 70–1000 *m/z*. Injection volume was 4 μL for negative ion mode and 2 μL for positive ion mode. The total run time per sample was 20.5 minutes. Probe (HESI probe) temperature was maintained at 350°C for both positive and negative run with a spray voltage of 3500 V and a capillary temperature of 320 °C [42].

#### Metabolite data analysis

Data tables with metabolite peaks (*mz*/*rt*) at 2-time points for consortium with drought stress and control drought stress treatment were formatted as comma separated values (.csv) files and uploaded to the MetaboAnalyst 3.0 server (http://www.metaboanalyst.ca) [51]. Metabolite data generated by UPLC-HRMS were checked for data integrity and normalized using MetaboAnalyst’s normalization protocols (selecting normalization by the sum, log transformation, and auto-scaling) to shrink any possible variance and to improve the performance for downstream statistical analysis.

Univariate analysis (t-test and one way ANOVA) was applied to calculate the statistical significance of the metabolites between 2 group means (consortium/drought). We applied multivariate methods, supervised method-Partial Least Squares Discriminant Analysis (PLS-DA) and unsupervised method-Hierarchical clustering with a heat map, for the comprehensive data analysis as it takes all the variables into consideration, for example, a heat map was generated based on the Pearson distance measure and the Ward clustering algorithm, showing the top 25 metabolites for consortium versus drought treatments by PLS-DA VIP (variable importance in projection) score using a significance level of *P* ≤ 0.05, and post-hoc analysis of Fisher’s LSD. The samples were arranged according to their sampling time points (time point 1 and 2) in all two groups. The important metabolites were identified by using 2 different methods separately: PLS-DA and SAM (Significant Analysis of Metabolites) [42, 52].

The pathway analysis was performed using MetaboAnalyst for the identified important metabolites using *Arabidopsis thaliana* pathway libraries. The Kyoto Encyclopedia of Genes and Genomes (KEGG) pathway database (http://www.genome.ad.jp/kegg/pathway.html) was also used for the metabolites that were not found in the *Arabidopsis* pathway libraries.

#### Data analysis for biochemical characters

The data analysis was carried out by using software Statistics, version. 8.1. An ANOVA was performed to determine the effect of treatments and error associated with the experiment. A total number of six replicates were used for each treatment. To identify significant differences among treatments, a mean comparison of traits was carried out by using protected LSD (*p* = 0.05) test where the error mean square was used to estimate the standard error of differences between mean.

## Results

### Chlorophyll content, chlorophyll fluorescence and relative water content (RWC) of two chickpea genotypes

Under drought condition, the tolerant genotype (93127) demonstrated higher chlorophyll content than the sensitive genotype (Punjab Noor-2009) (Table 1). The application of PGRs in combination with PGPRs (consortium) increased chlorophyll content in both genotypes, but the sensitive genotype was more responsive than tolerant genotype. The Fv/Fm ratio is an indicator of photosystem II damage in plants. The lower value indicates more damage to the photosystem. The sensitive genotype showed higher damage to the photosystem due to drought stress than the tolerant one. Both varieties showed responsiveness to the consortium treatment, but the sensitive genotype was more responsive than the tolerant variety. Regarding RWC, we also observed a similar trend and the sensitive genotype showed a higher increase in RWC compared to the tolerant genotype due to consortium treatment (Table 1). In summary, the consortium treatment increased chlorophyll content, Fv/Fm, and RWC in both genotypes compared to their drought treatment, but the increase was more in the sensitive genotype than the tolerant genotype. Consortium treatment helped to maintain the water balance in cell and to reduce damage to the photosystem in chickpeas.

**Table 1:** Chlorophyll content and chlorophyll florescence (Fv/Fm ratio) of two chickpea genotypes under consortium and drought condition after 25 days of stress imposition.

Chickpea genotype	Spad Chlorophyll Content		Chlorophyll Florescence (Fv/Fm)		RWC (%)		Protein (μg/g)		Sugar (mg/g)		Phenolics (mg GAE/g)		Shoot dry wt. (g)		Root dry wt. (g)	
	Cons.	Dro	Cons.	Dro.	Cons.	Dro.	Cons.	Dro.	Cons.	Dro.	Cons.	Dro.	Cons.	Dro.	Cons.	Dro.
Punjab Noor-2009 (Sensitive genotype, G1)	35.6±0.011^a^	16.4±0.01^b^	0.727±0.014^a^	0.317±0.015^b^	64±0.02^a^	27±0.07^b^	1.6±0.003^a^	1.2±0.016^b^	1.8±0.014^a^	0.7±0.001^b^	3.1±0.012^a^	1.7±0.013^b^	10.9±0.15^a^	3.11±0.1^b^	2.41±0.06^a^	0.94±0.03^b^
93127 (Tolerant genotype, G2)	46.1±0.021^a^	34.4±0.017^b^	0.843±0.007^a^	0.643±0.03^b^	79±0.011^a^	55±0.01^b^	1.7±0.015^a^	1.5±0.023^b^	2.3±0.019^a^	1.5±0.015^b^	3.7±0.08^a^	2.6±0.016^b^	12.27±0.28^a^	6.33±0.21^b^	2.4±0.08^a^	1.51±0.02^b^

**Cons-Consortium (Mean±SE); Dro-Drought (Mean±SE)**; Different letters (i.e. a and b) indicate significant differences (P< .05) among treatments.

### Protein, sugar and phenolics content in the leaves of two chickpea genotypes

The tolerant genotype showed a higher value for protein and phenolic compound under drought condition compared to the sensitive genotype (Table 1). However, the sugar content was higher in the sensitive genotype than the tolerant genotype under drought condition. Consortium treatment induced higher protein, sugar, and phenolic compound accumulation in both genotypes, however, the increase was more in the sensitive genotype than the tolerant one.

### Shoot and root dry weights

The tolerant genotype produced higher root and shoot dry weight than the sensitive genotype under drought condition (Table 1). Both genotypes showed increased root and shoot dry weight accumulation due to consortium treatment, and the increase was higher in the sensitive genotype than the tolerant one. In general, consortium treatment helped the plant to maintain growth by accumulating higher biomass under drought condition.

### Metabolic profile of chickpea genotypes induced by consortium and drought treatments

The untargeted UPLC-HRMS global metabolomics analysis was performed for profiling of leaf metabolites for 2 different chickpea genotypes under 2 contrasting treatments at 2 different sampling time points (14 and 25 days after stress imposition). UPLC-HRMS analysis detected a total of 178 known metabolite peaks out of which 53 were found to be significant (S1 Table). Metabolites were highly reproducible among the six analyzed biological replications at the 2 different time points.

#### PLS-DA and 2D loading plot results

**Fig 1:**
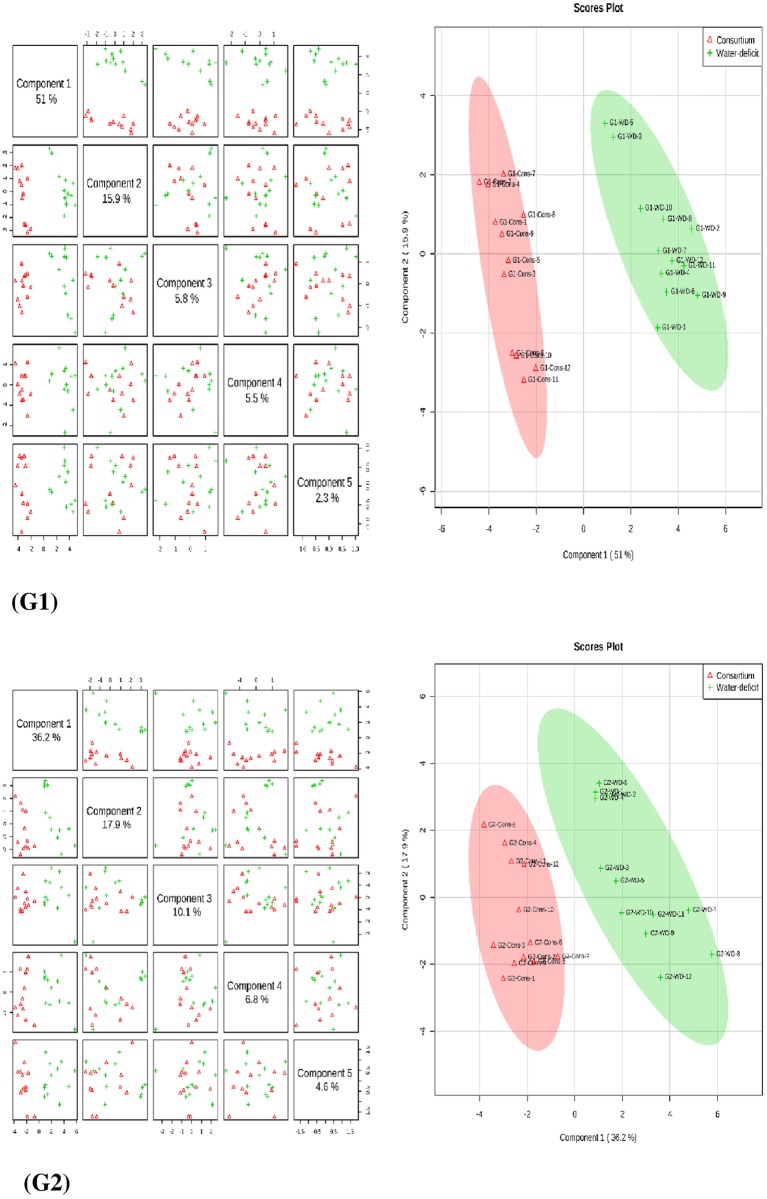
Partial least square discriminant analysis (PLS-DA) and 2D Scores loading plot for the chickpea Punjab Noor-2009 (G1) and 93127 (G2) leaves at 2 time points under consortium and water deficit treatments. Metabolites at consortium and drought treatments did not overlap indicating an altered state of metabolite levels in the chickpea leaves. Sampling time points are thereby demonstrating its effect over time and proofing in the leaves of chickpea plants.

**Fig 2:**
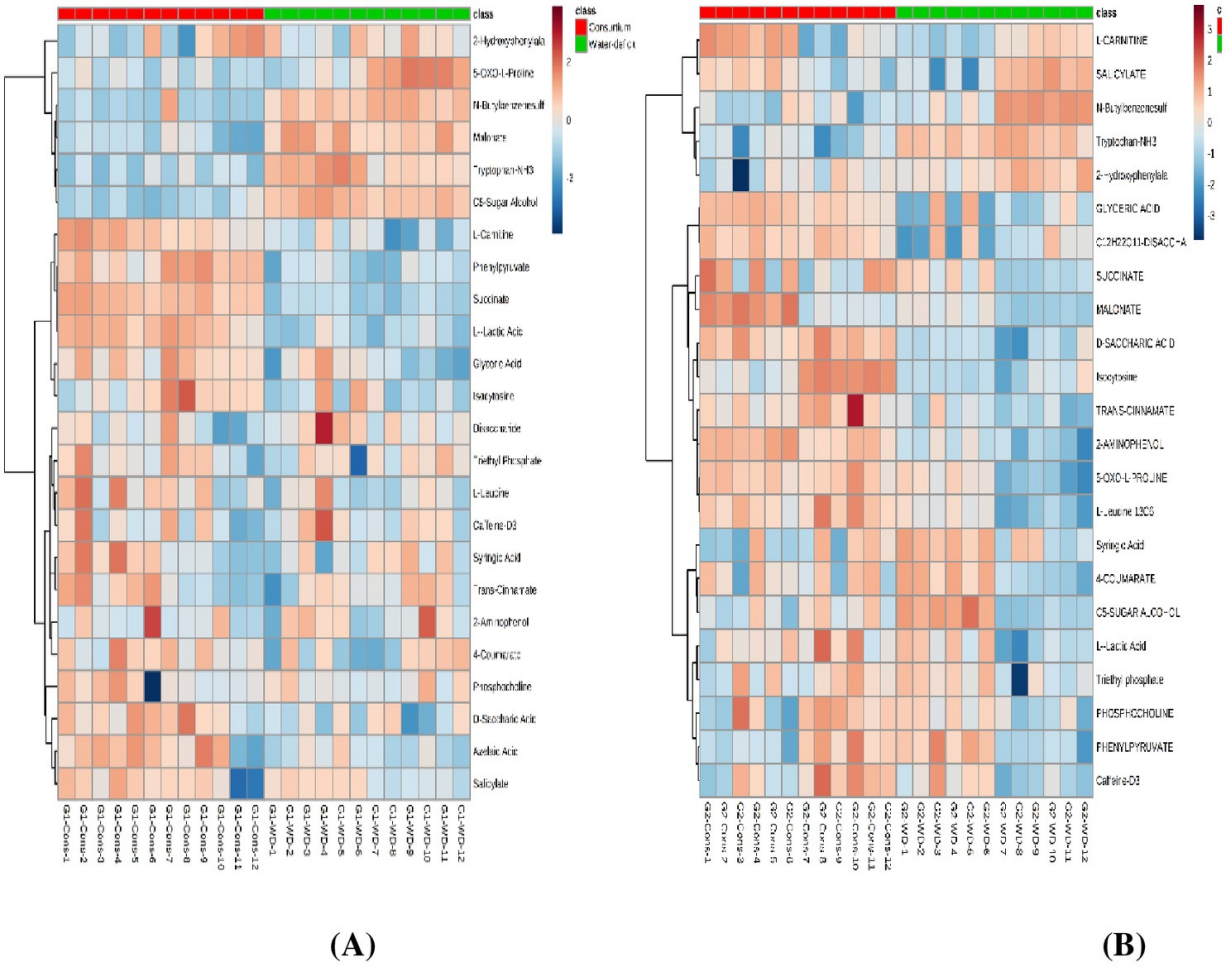
(A & B). Heatmap [A: consortium vs water-deficit (G1) and B: consortium vs water-deficit (G2)] illustrating (distance measure: Pearson; Clustering algorithm: Ward) of the performed partial least square discriminant analysis (PLS-DA) showing levels of key metabolite. Metabolite feature areas were normalized and range-scaled across all experimental samples at 2 different time points.

We performed a supervised clustering method, Partial Least Squares-Discriminant Analysis (PLS-DA), for both the genotypes and for consortium versus drought treatments at 2 different time points. PLS-components (PCs) analysis revealed that component 1 explained 51% and 36.2% of the total variation of the sensitive and tolerant genotypes, respectively, under consortium versus drought treatment (Fig 1), while the second component explained 15.9% and 17.9% variation for sensitive and tolerant genotypes, respectively, for the same treatment (Figs 1 and 2). The 2D scores plots between PC1 and PC2 showed 2 different groups associated with the consortium and drought samples at 2 different sampling points (Fig 1), suggesting a clear distinction in the metabolite accumulation under two different conditions. The separation between the consortium and the drought treatments is suggesting the dominant role of PGRs and PGPRs in modulating drought tolerance in treated plants.

#### Analysis of variance and heat map

A total of 53 metabolites were identified through a multi-factorial ANOVA which were significantly altered in 2 genotypes across 2 different time points and treatments. Among different groups of metabolites, amino acids, sugars, sugar alcohol, organic acids, polyamines, nitrogenous compounds and polyphenols and other organic compounds were significantly accumulated in the leaves of plants treated with PGRs + PGPRs under drought stress. Amino acid: leucine; organic compounds: succinate, lactic acid, phenylpyruvate, trans-cinnamate, 2-aminophenol, and malonate; sugar acid: glyceric acid; sugar: disaccharides; chemical compounds: saccharic acid, syringic acid; and ammonium compound: L-carnitine showed increased levels of accumulation in the leaves of plants treated with PGRs and PGPRs consortium. However, compounds like 5-oxo-proline tryptophan, 4-coumorate, sugar alcohol, salicylate, and phosphocholine were highly accumulated in the leaves of untreated plants grown under stress condition.

Significantly different metabolites (identified through ANOVA) were analyzed by hierarchical clustering with a heat map in order to visualize the effect of PGRs + PGPRs consortium on metabolomics expression over uninoculated drought stress plants. The heat map was generated for consortium versus drought treatments for both the sensitive and tolerant genotypes which indicates a clear distinction between metabolites at consortium and drought treatments. The first cluster of heat map was represented by metabolites accumulated at higher levels in the leaves of PGPRs + PGRs treated (consortium) plants including carnitine, glyceric acid, phenylpyruvate, succinate, lactic acid, leucine, D-saccharic acid, isocytosine and coumarate whereas, tryptophan, sugar alcohol, N-butylbezalsulfomide and malonate were abundantly present in leaves of untreated sensitive genotype grown under drought stress (Fig 2A). Accumulation of L-carnitine, salicylate, succinate, and malonate occurred only at the first time point in the leaves of PGRs + PGPRs treated plants of tolerant genotype whereas, isocytosine, trans-cinnamate, syringic acid, phosphocholine, and phenylpyruvate were only accumulated at second time point. The consortium of PGRs and PGPRs also significantly enhanced the accumulation of glyceric acid, disaccharide, saccharic acid, aminophenol and 5-oxo-L-proline at both time points in the tolerant genotype (Fig 2B).

#### Profiling of leaf metabolites

**Fig 3:**
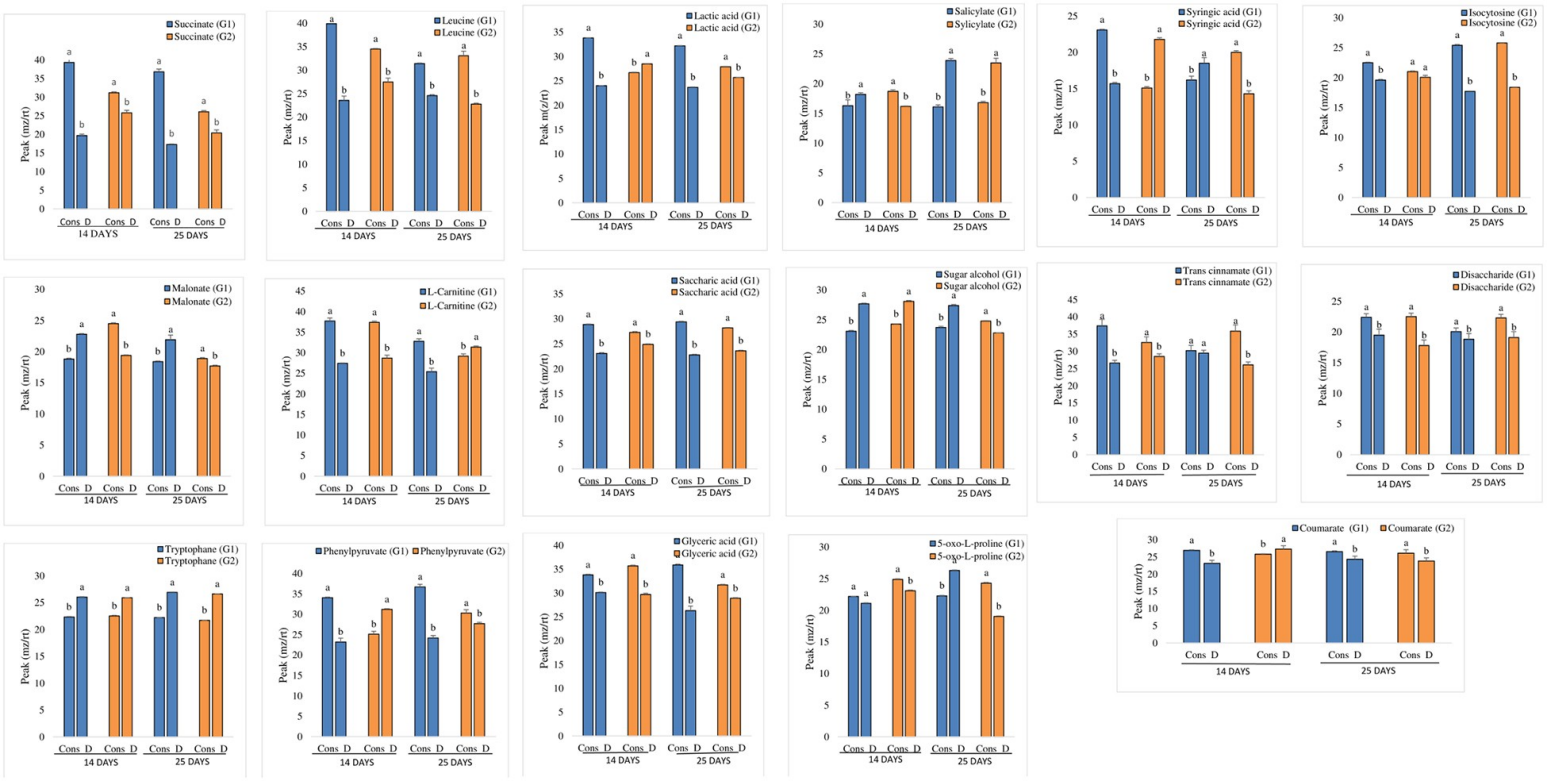
Significantly different levels of selected metabolites (ANOVA, *P* ≤ .05, Tukey’s honest significant difference) in the leaves of two chickpea varieties under consortium and drought conditions at 2 time points (14 and 25 days). G1: drought-sensitive chickpea genotype (Punjab Noor-2009); G2: drought-tolerant chickpea genotype (93127). Error bars represent standard errors of the mean (*n* = 6) at each time point. Cons = consortium, D = drought. Different letters indicate significant differences (*P* < .05) among treatments (consortium vs drought) for a genotype for *mz*/*rt* peak in a particular time point.

Two statistical methods, PLS-DA and SAM, were carried out for the identification of the most important metabolites across genotypes and treatments (Table 2). The PLS-DA analysis identified 25 most important metabolites based on the VIP score using a 5-component model. Similarly, the most important metabolites were also identified by significant analysis of metabolites (SAM) with the delta value of 1.5, false discord rate (FDR) of 0.002 with less than one (0.25) false positive. Overall, the identified metabolites were quite similar across the 2 methods. The top most important 25 metabolites which were identified by these 2 methods are shown in Table 2. These important metabolites included different amino acids, sugars, sugar alcohol, amines, organic acids, fatty acids and other intermediate compounds. The sensitive genotype significantly accumulated succinate, leucine, carnitine, lactic acid, glyceric acid, phenylpyruvate, isocytosine, and saccharic acid when treated with PGRs and PGPRs consortium (Fig 3). Contrary to that, compounds like malonate, disaccharide, and trans-cinnamate were highly accumulated in tolerant genotype under PGRs and PGPRs treatment. Drought stress caused significant accumulation of tryptophan, salicylate, and sugar alcohol in the leaves of untreated drought plants compare to the consortium in both tolerant and sensitive genotypes (Fig 3). More metabolites were significantly altered in sensitive genotype due to consortium treatment than tolerant genotype.

**Table 2:** Important metabolites with their compound ID (KEGG ID/PubChem CID and molecular formula, identified through partial least square discrepant analysis (PLS-DA) and significant analysis of metabolites (SAM) across genotypes and treatments.

S.No.	Important Metabolites	KEGG ID	PubChem CID	Molecular Formula	SAM (d-value)	PLS-DA VIP score (variance For component 1)
1	5-oxo-L-proline	C01877	107541	C_5_H_6_NO_3_	6.2298	0.10057
2	Azelaic acid	C08261	2266	C_9_H_16_O_4_	5.9834	0.4252
3	Glyceric acid	C00258	439194	C_3_H_6_O_4_	7.7543	0.73388
4	Succinate	C00042	1110	C_4_H_6_O_4_	9.4668	1.4221
5	L-(+)-Lactic Acid	C00186	107689	C_3_H_6_O_3_	4.961	1.3168
6	Phenylpyruvate	C00166	997	C_9_H_8_O_3_	3.854	1.2303
7	Choline	C00114	305	C_5_H_14_NO	5.936	-
8	Tryptophan-NH3	C00078	6305	C_11_H_12_N_2_O_2_	3.112	1.4373
9	L-Leucine 13C6	C00123	6106	C_6_H_13_NO_2_	-4.189	1.1682
10	Caffeine-D3	C07481	2519	C_8_H_**10**_**N**_**4**_O_2_	-4.532	1.224
11	2-Hydroxyphenylalanine	C00082	91482	C_9_H_11_NO_3_	2.524	0.5669
12	Syringic Acid	C10833	10742	C_9_H_10_O_5_	5.265	0.86745
13	Trans-cinnamate	C00423	444539	C_9_H_8_O_2_	4.341	0.62645
14	D-Saccharic acid	C00818	33037	C_6_H_10_O_8_	3.265	1.1957
15	Triethyl phosphate	-	6535	C_6_H_15_O_4_P	1.474	1.0598
16	L-Carnitine	C00318	2724480	C_7_H_15_NO_3_	7.284	1.1691
17	2-Aminophenol	C01987	23035081	C_6_H_7_NO	2.193	0.75572
18	N-Butylbenzenesulfonamide	-	19241	C_10_H_15_NO_2_S	1.743	1.0977
19	Isocytosine	-	66950	C_4_H_5_N_3_O	4.983	0.74784
20	4-Coumarate	C00811	637542	C9H8O3	8.974	1.1368
21	Malonate	C00383	867	C_3_H_4_O_4_	5.846	1.0699
22	Salicylate	C07588	10253	C_7_H_6_O_3_	3.957	0.70754
23	C5-Sugar alcohol	-	-	-	3.654	0.92692
24	Disaccharide	C00089	5988	C_12_H_22_O_11_	2.884	0.76615
25	Phosphocholine	C00588	1014	C_5_H_15_NO_4_P+	2.631	0.38416

KEGG = Kyoto Encyclopedia of Genes and Genomes.

#### Identification of corresponding biological pathways

All the metabolites significantly affected by PGRs + PGPRs treatment under drought stress were mapped to the biological pathways involved in the KEGG online database, which was assigned to 22 different pathways/metabolism in either treatment. Table 3 is showing the metabolites involved in each pathway, the number of hits, and FDR of the pathway. As expected, the significantly altered metabolites were involved in a number of different pathways. These include Phenylalanine, tyrosine and tryptophan biosynthesis, Phenylpropanoid biosynthesis, Glucosinolate biosynthesis, Tropane, piperidine and pyridine alkaloid biosynthesis, Citrate cycle (TCA cycle), Aminoacyl-tRNA biosynthesis, Ubiquinone and terpenoid-quinone biosynthesis, Glycolysis or Gluconeogenesis, Valine, leucine and isoleucine degradation, and Valine, leucine, and isoleucine biosynthesis. In addition, 11 primary metabolisms such as the Phenylalanine metabolism, Ascorbate and aldarate metabolism, Glycerolipid metabolism, Propanoate metabolism, Glyoxylate and dicarboxylate metabolism, Tyrosine metabolism, Pyruvate metabolism, Butanoate metabolism, Alanine, aspartate and glutamate metabolism, Glycerophospholipid metabolism, Tryptophan metabolism and Glycine, serine and threonine metabolism were also significantly altered due to consortium and drought treatments.

**Table 3:** Pathway names, total metabolites involved in that pathways, metabolites significantly accumulated in present study (hits), and false discord rate (FDR).

Pathway name	Total	Hits	FDR
Phenylalanine metabolism	11	3	2.2225E-5
Phenylalanine, tyrosine and tryptophan biosynthesis	22	2	0.000108
Glycerophospholipid metabolism	25	2	0.000109
Phenylpropanoid biosynthesis	31	2	0.00369
Glucosinolate biosynthesis	8	1	0.00387
Tropane, piperidine and pyridine alkaloid biosynthesis	10	1	0.00525
Ascorbate and aldarate metabolism	14	1	0.00574
Glycerolipid metabolism	14	1	0.00656
Propanoate metabolism	14	1	0.00793
Glyoxylate and dicarboxylate metabolism	17	1	0.00999
Tyrosine metabolism	18	1	0.01001
Pyruvate metabolism	20	1	0.01436
Butanoate metabolism	20	1	0.01446
Citrate cycle (TCA cycle)	20	1	0.01563
Alanine, aspartate and glutamate metabolism	21	1	0.01612
Aminoacyl-tRNA biosynthesis	67	2	0.01801
Ubiquinone and other terpenoid-quinone biosynthesis	22	1	0.01823
Glycolysis or Gluconeogenesis	25	1	0.01893
Tryptophan metabolism	25	1	0.0221
Valine, leucine and isoleucine biosynthesis	26	1	0.0251
Glycine, serine and threonine metabolism	29	1	0.0325
Valine, leucine and isoleucine degradation	34	1	0.0471

## Discussion

Drought stress is one of the major constraints for agricultural productivity throughout the world. Approximately, 40% of the agricultural lands are located in the arid and semi-arid regions of the world [53] where plants suffer frequently from drought stress. Both plant hormones (PGRs) and PGPRs respond to environmental stresses and impart tolerance to plant against the stresses [54]. PGRs and PGPRs consortium plays a significant role in the alleviation of drought stress in plants by maintaining water budget of the plant and by producing metabolites as observed during the present investigation. In the sensitive genotype, the ameliorating effect of PGRs and PGPRs consortium was noteworthy. Chlorophyll content, chlorophyll fluorescence, RWC, and root and shoot biomass accumulation were greater than 2 fold due to PGRs and PGPRs consortium under drought stress. The effect of PGRs and PGPRs treatment was more pronounced in the sensitive genotype compared to the tolerant genotype. Previous studies demonstrated that the drought-induced reduction in chlorophyll content and chlorophyll fluorescence led to a decrease in photosynthesis and overall plant growth [55, 56]. The combined treatment significantly enhanced the chlorophyll fluorescence and chlorophyll content values in the PGRs/PGPRs treated plants in both genotypes. The application of PGRs significantly enhanced the root and shoot growth, and dry matter production in different plants [42, 57]. Similarly, PGPRs are known to enhance the root growth and uptake of minerals and water, thus promote the growth of the whole plant which in turn has a positive impact on plant dry matter content in wheat [58] and in chickpea [59]. Hassanzadeh et al. [60] reported that decrease in RWC is related to the decrease in chlorophyll content and leaf fresh weight in sesame genotypes, however, the tolerant genotypes maintained higher RWC under stress condition and thus showed higher affinity for chlorophyll content and leaf fresh weight. Our results demonstrated that PGRs combined with PGPRs helped both genotypes to maintain an efficient photosystem with improved water budget resulting in improved growth and productivity under drought stress condition.

Significant increase in the leaf protein content in both genotypes was evident after being treated with PGRs and PGPRs in comparison to untreated plants grown under stress condition. A correlation between increased protein levels and their involvement in ROS scavenging and oxidative stress metabolism have been demonstrated in plants under stress [61]. The higher protein accumulation was associated with significant increase in Fv/Fm and chlorophyll content due to PGRs and PGPRs treatment under drought stress condition. The increased level of proteins due to PGRs and PGPRs might have assisted plants to mitigate ROS effect [62, 63].

Under stress conditions, the leaf sugar content decreases significantly which is an indication of rapid senescence due to stress. PGRs in combination with PGPRs effectively increased the leaf sugar content under drought condition in both genotypes. Soluble sugars have been demonstrated to play an important role in responses to biotic and abiotic stresses [64]. Sugar signalling pathways interact with stress pathways into a complex network in plants to modulate metabolic responses [65]. Soluble sugars may either act directly as negative signals or as modulators of plant sensitivity and thus, they can also play important roles in cell responses to stress-induced remote signals [66]. Under stress conditions, a decrease in dry matter accumulation and depletion of sugar was correlated in the plant [67]. Present findings demonstrated an increased dry matter accumulation due to PGRs and PGPRs treatment under drought condition which could potentially be attributed to the better cellular osmotic balance (demonstrated by RWC) in photosynthetic organs, and thus helped to maintain higher photosynthetic rate and growth. PGRs and PGPRs treatments have also increased the production of phenolic compounds. These molecules have been described as markers for abiotic stress tolerance in plants [68] and they have been proclaimed to be involved in oxidative stress caused by ROS [69].

UPLC-HRMS based untargeted metabolic profiling in the leaves of two chickpea genotypes was performed to understand the effect of PGRs and PGPRs on metabolic changes to adjust drought stress condition. The increased accumulations of different metabolites were previously reported under drought condition in different plant species [42, 70, 71]. It has been noted that the accumulation of leucine, succinate, lactic acid, and glyceric acid was higher in the sensitive genotype at both time points when treated with PGRs + PGPRs consortium. Leucine and other amino acids are known to play a variety of different roles in plants, especially under stress condition and impart drought tolerance [72]. Previously, we have reported the increased level of amino acids and organic acids in tolerant variety grown under drought condition as compared to control plants [42]. Coupled with different amino acids, our study also demonstrated an increased level of total protein accumulation due to PGRs and PGPR treatment. The increased level of different amino acids and protein were associated with water balance, intact photosynthetic structure, and high biomass accumulation in chickpeas plants treated with PGRs + PGPR consortium in our study.

Significant accumulation of L-carnitine, trans-cinnamate, succinate and syringic acid occurred at the first time point, whereas, saccharic acid, isocytosine, hydroxyphenylalanine and phenylpyruvate showed significant accumulation in the leaves of PGRs + PGPRs treated sensitive genotype at the second time point. L-carnitine regulates the level of acyl-CoA and CoA in the mitochondrion and cytosol and involvs in the regulation of water resorption and photosynthesis [73]. Plants produce a moiety of organic compounds in response to a variety of environmental stimuli which have key ecological functions and involved in interactions with biotic and abiotic stresses. These compounds are responsible for osmoregulation in both plants and animals. Cinnamate and coumarate are widely distributed in the plant kingdom and play a key role in plant defence, growth and plant-insect interactions [74]. Succinate act as a primary intermediate in ATP pathway of Kreb cycle and play a vital role in energy production and regulation of mitochondrial TCA cycle [75]. Excess of succinate in plants under stress results in more ATP production in mitochondria [76–78]. The elevated level of succinate found in PGRs and PGPRs treated plants demonstrated better tolerance to drought stress and this could potentially be attributed to the efficient TCA cycle that produces more energy under water-limited conditions. It was evident that all these compounds were involved in different biologically significant activities, such as osmoregulation, photosynthesis, energy production or defense activity in the plant. Their biological functions correlate with our study through osmoregulation or increased photosynthetic efficiency or through higher biomass accumulation in chickpea plants treated with PGRs and PGPRs under drought stress conditions.

The present study has demonstrated enhanced accumulation of disaccharide, saccharic acid, glyceric acid, aminophenol, and 5-oxo-L-proline at both time points in the tolerant genotype when treated with PGRs and PGPRs consortium. These sugars are responsible for osmotic adjustment by detoxifying reactive oxygen species and stabilize the quaternary structure of protein under water scarcity [79]. Accumulation of sugars and their derivatives lead to drought tolerance in wheat, maize, Arabidopsis, chickpea, millet and rye [42, 80, 81]. Higher accumulation of the total sugar content was also evidenced in our study due to PGRs and PGPRs treatment which was due to increased photosynthetic activity. Higher accumulation of sugar alcohol was noted in drought-tolerant genotype treated with PGRs + PGPRs. Sugar alcohols also play a significant role in stress tolerance by inducing osmotic adjustment through accumulation of a compatible solute or the transitory storage of carbon reserves [82].

The untreated genotypes exhibited a higher accumulation of salicylate and tryptophan when exposed to long-term drought stress. In plants, exogenous application of salicylates affected many physiological and biochemical processes such as seed germination, seedling establishment, thermogenesis cell growth, senescence, stomatal responses, thermotolerance and nodulation [83–85]. Reduced accumulation of salicylates due to PGRs and PGPR consortium treatment could potentially be attributed to a reduced rate of senescence and thus prolong the photoassimilation. Tryptophan plays a major role in the regulation of plant development and defense responses [86]. Tryptophan is the precursors of different secondary metabolites including indoleacetate, lipid precursor, and lignin in the Shikimate pathway, which plays a vital role in stress tolerance [87].

The link between different metabolic pathways and associated metabolites was stimulated using MetaboAnalyst. Twenty five metabolic pathways were significantly altered using the Kyoto Encyclopedia of Genes and Genomes database (KEGG) and *Arabidopsis* annotation project database. The Phenylalanine, tyrosine and tryptophan biosynthesis pathway was upregulated in the PGRs and PGPRs treated plants. This is an important pathway for the synthesis of essential aromatic amino acids. These aromatic amino acids not only serve as part of protein biosynthesis but also involved in the synthesis of other important secondary metabolites that play key roles in plant growth and development [88, 89]. Aminoacyl-tRNA biosynthesis and citrate cycle was also altered in the present study due to PGRs and PGPRs treatment. Aminoacyl-tRNA biosynthesis is a group of twenty different enzymes that establish the rules of genetic code. It had been reported earlier that the disrupted metabolic conditions is associated to a specific aminoacyl-tRNA synthetase [90]. The aminoacyl-tRNA synthetases catalyse the binding of amino acids to their specific tRNA and thus play a key role in translation and in gene expression. Citrate cycle play a key role in producing ATP and providing carbon skeletons for a variety of biosynthetic processes in both heterotrophic and photosynthetic tissues [91]. Glycine, serine and threonine metabolism play an important role during signalling process and in plant stress responses [92]. Glycine and Serine are two interconvertible amino acids that play significant role in C1 metabolism. Whereas, Serine has a central role in the metabolism and signalling, and involved in plant homeostasis.

## Conclusion

Different amino acids, sugars, sugar alcohol, amines, organic acids, fatty acids and other intermediate compounds were changed significantly due to PGRs and PGPRs treatment. Similar to physiological responses, sensitive genotype also showed altered levels of more metabolites than tolerant genotype. The accumulation of succinate, leucine, disaccharide, saccharic aid and glyceric acid was significantly higher in both genotypes in both time points due to PGRs and PGPRs treatment. As these metabolite levels were constantly higher in both genotypes and at different time points, demonstrating their roles in monitoring biochemical pathways related to drought tolerance. Significant accumulation of malonate, 5-oxo-L-proline, and trans-cinnamate occurred at both time points only in the tolerant genotype due to the consortium treatment. On the contrary, lactic acid, L-carnitine, isocytosine, and phenylpyruvate were accumulated significantly in sensitive genotypes at both times. These results indicate that the higher accumulation of these metabolites could possibly associated only with the tolerance mechanism in sensitive genotype. These data provide information that may, with further investigation, help to understand the biochemical pathway underlying drought stress tolerance in chickpea induced by PGRs and PGPRs treatment.

## Supporting information

S1 Table List of top 53 significant metabolites identified in the study with their compound type, identifier (KEGG ID/PubChem CID*), molecular formula, P-value and false discovery rate (FDR), mass-to-charge ratio (m/z), and retention time (RT). (KEGG = Kyoto Encyclopedia of Genes and Genomes).
(DOCX)
