# Supplementary material for: Metabolic and physiological changes induced by plant growth regulators and plant growth promoting rhizobacteria and their impact on drought tolerance in Cicer arietinum L
Source: PLoS One. 2019 Mar 4;14(3):e0213040. doi: 10.1371/journal.pone.0213040 (PMC6398973; doi:10.1371/journal.pone.0213040)
Supplement: S1 Table — (KEGG = Kyoto Encyclopedia of Genes and Genomes). (DOCX) [file pone.0213040.s001.docx]

**S1 Table.** List of top 53 significant metabolites identified in the study with their compound type, identifier (KEGG ID/PubChem CID*), molecular formula, P-value and false discovery rate (FDR), mass-to-charge ratio (m/z), and retention time (RT). (KEGG=Kyoto Encyclopedia of Genes and Genomes).

| **S.no** | **Compound Name** | **Compound type** | **Compound ID** | **Molecular formula** | **p-value** | **FDR** | **M/Z value** | **RT value** |
| --- | --- | --- | --- | --- | --- | --- | --- | --- |
| 1 | Choline | Essential nutrient | C00114 | C5H13NO | 5.30E-10 | 2.34E-07 | 104.1 | 0.825 |
| 2 | Phenylalanine | Amino acid | C00079 | C9H11NO2 | 2.75E-06 | 3.41E-07 | 166.09 | 6.286 |
| 3 | 5-Oxo-L-Proline | Amino acid | C01879 | C5H7NO3 | 4.45E-10 | 7.43E-10 | 130.05 | 1.766 |
| 4 | L-Serine | Amino acod | C00065 | C3H7NO3 | 2.24E-05 | 4.14E-05 | 106.05 | 0.7179 |
| 5 | Riboflavin | Vitamin | C00255 | C17H20N406 | 0.000112 | 0.000153 | 377.14 | 7.9834 |
| 6 | Tryptophan | Amino acid | C00078 | C11H12N202 | 3.14E-10 | 3.13E-05 | 205.1 | 7.648 |
| 7 | Allantoin | Chemical Compound | C02350 | C4H6N4O3 | 1.12E-06 | 1.99E-09 | 157.04 | 0.804 |
| 8 | Phenylpyruvate | Organic compound | C00166 | C9H8O3 | 4.47E-10 | 3.34E-09 | 163.04 | 7,791 |
| 9 | Succinate | Dicarboxylic acid | C00258 | C4H6O4 | 3.64E-11 | 1.52E-09 | 117.02 | 2.27 |
| 10 | Threonine | Amino acid | C00188 | C4H9NO3 | 2.15E-07 | 6.58E-06 | 120.07 | 0.725 |
| 11 | Phosphocholine | Intermediate compound | C00588 | C5H15NO4P+ | 5.97E-10 | 1.17E-03 | 184.07 | 0.7249 |
| 12 | Tryptophan-NH3 | Amino acid | C00158 | C11H10NO2 | 1.04E-10 | 2.89E-07 | 188.07 | 7.649 |
| 13 | Azelaic acid | Organic compound | C08261 | C9H16O4 | 5.77E-10 | 3.93E-09 | 187.1 | 8.486 |
| 14 | Glyceric acid | Sugar acid | C00258 | C3H6O4 | 4.24E-10 | 2.31E-08 | 105.02 | 0.828 |
| 15 | L-Leucine 13C6 | Amino acid | C00123 | C6H13NO2 | 1.77E-10 | 1.44E-03 | 132.1 | 2.322 |
| 16 | Caffeine-D3 | Stimulator | C07481 | C8H10N4O2 | 2.51E-08 | 4.66E-04 | 198.1 | 7.878 |
| 17 | 4-Aminobutanoate (GABA) | Chemical compound | C00334 | C4H9N02 | 2.47E-04 | 5.42E-04 | 104.07 | 0.796 |
| 18 | Nicotinamide | Vitamin | C00153 | C6H6N2O | 0.003244 | 2.48E-03 | 123.05 | 1.561 |
| 19 | L-(+)-Lactic Acid | Organic compound | C00186 | C3H603 | 1.62E-10 | 3.38E-09 | 91.04 | 0.8321 |
| 20 | Guanine | Nucleobase | C00242 | C5H5N5O | 0.00439 | 3.63E-05 | 152.06 | 1.4405 |
| 21 | 2-Hydroxyphenylalanine | Metabolite | 91482* | C9H11NO3 | 1.84E-07 | 4.43E-04 | 182.08 | 3.4135 |
| 22 | Alanine | Amino acid | C00041 | C3H7NO2 | 2.43E-14 | 4.39E-13 | 90.06 | 0.714 |
| 23 | Aspartate | Amino acid | C00049 | C4H7NO4 | 0.00543 | 2.54E-02 | 134.04 | 0.718 |
| 24 | BOC-L-Tyrosine | Amino acid | 117439* | C14H19NO5 | 2.34E-06 | 3.97E-08 | 182.08 | 10.003 |
| 25 | 4-Acetamidobutanoate | Amino acid | C02946 | C6H10NO3 | 0.004744 | 0.00115 | 144.07 | 3.46 |
| 26 | Glycerophosphocholine | Natural choline | C00670 | C8H20NO6P | 0.00549 | 0.003024 | 258.1 | 0.748 |
| 27 | Disaccharide | Sugar | C00089 | C12H22O11 | 0.000213 | 6.41E-04 | 341.1 | 0.857 |
| 28 | Dopamine | Organic chemical | C03758 | C8H11NO2 | 0.005534 | 0.004231 | 154.08 | 2.679 |
| 29 | Phosphocholine | Vitamin | C00588 | C5H14NO4P | 0.003362 | 1.13E-05 | 184.07 | 0.7249 |
| 30 | 3-Hydroxy-3-Methylglutarate | Dicarboxalic acid | C03761 | C6H10O5 | 0.053421 | 0.004354 | 185.04 | 2.9513 |
| 31 | Erythritol | Sugar alcohol | C00503 | C4H10O4 | 0.000423 | 0.000432 | 145.05 | 0.8454 |
| 32 | Glucosamine | Amino sugar | C00329 | C6H13NO5 | 0.000283 | 0.000533 | 180.09 | 0.7441 |
| 33 | Syringic Acid | Trihydroxybenzoic acid | C10833 | C9H10O5 | 3.17E-10 | 5.28E-09 | 199.05 | 7.419 |
| 34 | Trans-cinnamate | Organic compound | C00423 | C9H8O2 | 5.07E-10 | 3.91E-06 | 149.05 | 9.761 |
| 35 | D-Saccharic acid | Chemical compound | C00818 | C6H10O8 | 3.55E-10 | 5.03E-07 | 209.03 | 0.736 |
| 36 | Triethyl phosphate | Chemical compound | 6535* | C6H15O4P | 0.00363 | 0.000423 | 183.07 | 9.322 |
| 37 | L-Carnitine | Ammonium compound | C00318 | C7H15NO3 | 2.03E-10 | 3.11E-05 | 162.1 | 1.154 |
| 38 | 2-Aminophenol | Organic compound | C01987 | C6H7NO | 1.16E-10 | 0.005342 | 110.06 | 1.894 |
| 39 | N-Butylbenzenesulfonamide | Chemical compound | 19241* | C10H15NO2S | 0.00353 | 0.004512 | 214.08 | 11.889 |
| 40 | N-Methyl-L-Glutamate | Chemical compound | C01046 | C6H11NO4 | 4.31E-07 | 5.28E-06 | 162.08 | 1.0811 |
| 41 | Dibutyl-phthalate | Organic compound | C03690 | C16H22O4 | 0.00142 | 0.000701 | 279.16 | 14.71997 |
| 42 | L-Pipecolic Acid | Organic molecule | C00408 | C6H11NO2 | 0.000413 | 0.000424 | 130.09 | 1.209 |
| 43 | Cytidine | Nucleoside | C00475 | C9H13N3O5 | 0.00363 | 0.000634 | 242.08 | 1.608 |
| 44 | Ascorbic Acid | Vitamin | C00072 | C6H8O6 | 0.00532 | 1.82E-03 | 175.02 | 1.005 |
| 45 | Isocytosine | Pyrimidine base | 66950* | C4H5N3O | 2.22E-10 | 3.06E-06 | 112.05 | 1.6 |
| 46 | 4-Coumarate | Chemical compound | C00811 | C9H8O3 | 5.32E-10 | 5.49E-08 | 163.04 | 8.231 |
| 47 | Malonate | Dicaroboxylic acid | C00383 | C3H4O4 | 1.52E-10 | 3.54E-05 | 103 | 1.126 |
| 48 | Salicylate | Phenolic acid | C07588 | C7H6O3 | 2.74E-10 | 4.01E-08 | 137.02 | 9.346 |
| 49 | 5-Aminolevulinic Acid | Non protein amino acid | C00430 | C5H9NO3 | 0.03325 | 0.005242 | 132.07 | 0.7299 |
| 50 | Glutamate | Organic compound | C00025 | C5H8O4 | 0.032423 | 0.003515 | 147.08 | 0.722 |
| 51 | Tartaric Acid | Organic acid | C00898 | C4H6O6 | 0.023958 | 0.002753 | 149.01 | 0.773 |
| 52 | Glycerol | Polyol | C00116 | C3H8O3 | 0.00074328 | 0.02643 | 115.04 | 0.794 |
| 53 | Uracil | Nucleobase | C00106 | C4H4N2O2 | 0.009228 | 0.020086 | 113.03 | 1.3989 |
